# A Novel mHealth Monitoring System during Cycling in Elite Athletes

**DOI:** 10.3390/ijerph18094788

**Published:** 2021-04-30

**Authors:** Alexandros Iliadis, Milena Tomovic, Dimitrios Dervas, Markella Psymarnou, Kosmas Christoulas, Evangelia Joseph Kouidi, Asterios Pantazis Deligiannis

**Affiliations:** 1 Sports Medicine Laboratory, Department of Physical Education and Sports Science, Aristotle University of Thessaloniki, 57001 Thessaloniki, Greece; alexcyclist@hotmail.com (A.I.); milenatomovic83@gmail.com (M.T.); dimdervas@gmail.com (D.D.); kouidi@phed.auth.gr (E.J.K.); 2 Vidavo S.A., Balkan Center, 57001 Thessaloniki, Greece; markella@vidavo.eu; 3 Laboratory of Evaluation of Human Biological Performance, Department of Physical Education and Sports Science, Aristotle University of Thessaloniki, 57001 Thessaloniki, Greece; kchristo@phed.auth.gr

**Keywords:** mHealth, tele-monitoring, cycling, health disorders

## Abstract

Background: Cycling is a very demanding physical activity that may create various health disorders during an athlete’s career. Recently, smart mobile and wearable technologies have been used to monitor physiological responses and possible disturbances during physical activity. Thus, the application of mHealth methods in sports poses a challenge today. This study used a novel mobile-Health method to monitor athletes’ physiological responses and to detect health disorders early during cycling in elite athletes. Methods: Sixteen high-level cyclists participated in this study, which included a series of measurements in the laboratory; health and performance assessments; and then application in the field of mHealth monitoring in two training seasons, at the beginning of their training period and in the race season. A field monitoring test took place during 30 min of uphill cycling with the participant’s heart rate at the ventilatory threshold. During monitoring periods, heart rate, oxygen saturation, respiratory rate, and electrocardiogram were monitored via the mHealth system. Moreover, the SpO_2_ was estimated continuously, and the symptoms during effort were reported. Results: A significant correlation was found between the symptoms reported by the athletes in the two field tests and the findings recorded with the application of the mHealth monitoring method. However, from the pre-participation screening in the laboratory and from the spiroergometric tests, no abnormal findings were detected that were to blame for the appearance of the symptoms. Conclusions: The application of mHealth monitoring during competitive cycling is a very useful method for the early recording of cardiac and other health disorders of athletes, whose untimely evaluation could lead to unforeseen events.

## 1. Introduction

Acute health disorders, such as musculoskeletal injuries, cardiovascular events, etc., may occur during long-distance competitive cycling due to prolonged strenuous exercise and sometimes environmental conditions [1]. Health disorders are more common in young athletes and particularly at the beginning of the training season [2]. This highly demanding sport discipline often leads to dehydration that can be further followed by hypotension; decreased coordination; fatigue; and, in some cases, fainting episodes [3]. More severe events attributed to cycling include arrhythmias, hypoxia, hypoglycemia, hyperventilation, and inappropriate dyspnea [4,5,6,7,8,9].

During a sports competition in real time, e-health monitoring is beneficial for determining the athlete’s response to the load of the exercise; assessing fatigue; and minimizing the risk of injury, cardiac problems, and other disorders [10,11]. Conventional measures of exercise load include power output, speed, time-motion analysis, global positioning system (GPS) parameters, and accelerometer-derived parameters [11,12]. Moreover, monitoring some hemodynamic responses, the cardiac rhythm, respiratory and metabolic indices, body temperature, and other settings is essential for safe exercise [10,11]. Smart mobile devices and wearable technologies are becoming increasingly useful for monitoring athletes’ physical activity and health disorders in sports. The World Health Organization has defined mHealth as the “use of mobile and wireless technologies to support the achievement of health objectives”. Mobile health (mHealth)-related applications are popular in health and fitness, according to recent studies [13,14]. However, there is a lack of continuous and comprehensive measurement of physiological parameters during cycling by such methods in elite athletes [15]. The application of such methods in competitive cycling is a challenge because of the sport’s characteristics.

The present study aimed to evaluate the application of a novel mobile telemetric procedure to monitor elite cyclists’ physiological parameters during strenuous cycling to detect health disorders that may appear or evolve during exercise in two training periods.

## 2. Materials and Methods

Sixteen high-level male cyclists aged 18–33 years from cycling clubs in Northern Greece with the best ranking participated in the study. Cyclists are ranked by the Greek Cycling Federation according to their position in domestic competitions (national and local championships, cups, inter-club competitions, cycling rounds) and abroad (Olympic Games and world championships, international rounds, Mediterranean and Balkan championships) on track, road, and mountain terrain. The design of the study included a series of measurements in the laboratory and then the application of the mHealth monitoring method in the field in two training seasons: firstly, at the beginning of the training cycle (November), and secondly at the end of the preparation period (during competition season—May and June). All measurements were taken in the morning (between 09:00 and 11:00 a.m.), while athletes abstained from alcohol and coffee at least 24 h before the tests. All the athletes were healthy, as evidenced by their history and the pre-participation health screening that took place in the Sports Medicine Laboratory of the Aristotle University of Thessaloniki; were not taking any medications; and completed the informed consent form. The University Ethics Committee approved the study protocol following the Helsinki Declaration for human research.

### 2.1. Laboratory Measurements

Participants were asked to fill in a pre-participation medical history questionnaire (including demographic, training, personal, and family medical history data). Then, anthropometric measurements (weight and BMI by FORA TN’G, Moorpark, CA, USA) and physical examinations were performed. Moreover, glucose measurement (Easy2Check Card Guard) and resting 12-lead electrocardiograms (ECG-PMP SelfCheck ECG Card Guard, Rehovot, Israel) were carried out. Finally, a maximal cardiopulmonary exercise test (CPET) via a breath-by-breath gas analyzing system (Ultima Series Med Graphics, Saint Paul, MN, USA) was contacted on a Seca Cardiotest 100 cycle ergometer, (Vogel & Halke Gmbh & Co, Hamburg, Germany). They performed the following maximal ramp exercise protocol until exhaustion: preheating (5 min at 100 W and 3 min at 150 W); test (3 min at 200 W, 3 min at 250 W); and then an increase of 25 W every minute to exhaustion. The pedaling speeds used were 80 to 90 ramp per minute. The athletes’ maximal oxygen consumption (VO_2_ max), maximal heart rate (HR max), and ventilator threshold (VT) were obtained. The HR at the VT point (HRVT) was also detected. All the pre-participation data were transferred to the previously generated electronic file for each cyclist. The exercise test was repeated before the second monitoring procedure.

### 2.2. mHealth Monitoring Procedure

**Figure 1:**
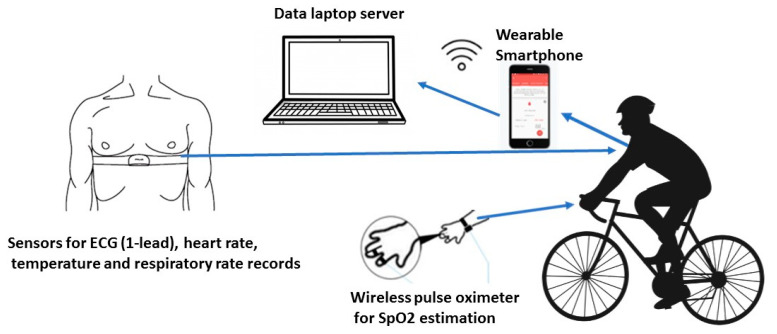
mHealth monitoring methodology.

The e-health system architecture, provided by Vidavo SA, a Greek e-Health company, was composed of four parts (Figure 1): (1) four sensors, suitable for a secure biomedical wireless transmission, mounted in a specific wearable belt (Vidavo SA, Zephyr BioHarness, Annapolis, MD, USA), placed on each cyclist’s chest under the papilla; (2) a receptor on-board to a Smartphone, where an embedded software allowed the pre-processing of acquired body sensor data. The Android Studio, which was the official IDE for Android development, and included everything someone needed to build Android apps, was used to build the software. In addition, some features were designed: the acquisition of data via Bluetooth, view of these data, control of the sensors, signaling the presence of noise; (3) a mobile Smartphone (Google Nexus S Android 4.0 operating system, Mountain View, CA, USA), which had GPS and Bluetooth connection managing the real-time data transmitting with the receptor and a GSM for real time data transmitting to the server; (4) a Linux based data server (laptop computer—Samsung Galaxy Book, Suwon, South Korea) oriented to store, process, and manage all data. Pilot studies of the mHealth system were performed before the study to check its reliability.

Within a week after the pre-participation examination, the cyclists were tested in the field. The field training test included 30 min of strenuous uphill cycling at each athlete’s HRVT after a warm up of 10 min of low-intensity straight path cycling. Perceived exertion, using Borg scale, speed, cadence, and heart-rate measurement via a monitoring system, was used to measure their effort during field training to maintain the desired level of cycling intensity. During cycling, each athlete’s ECG (1-lead), heart rate, temperature, and respiratory rate were recorded via the sensors. Simultaneously, SpO2 was recorded by a wireless pulse oximeter (OxyPro, Card Guard, Neuhausen am Rheinfall, Switzerland) placed on each athlete’s wrist and connected to the cover on the top of the finger. While recording, all available data were automatically transferred through the mobile phone, which was in the pocket of every cyclist, to the car escort’s laptop and stored in the electronic file of each cyclist. Moreover, at rest and at the end of each monitoring test, blood pressure (Omron M7 Intelli IT Comfort, Hoofddorp, Netherlands), glucose (Easy2Check Card Guard, Rehovot, Israel), and hemoglobin levels (Hemosmart Apex Biotechnology Corporation, Hsinchu City, Taiwan) were measured and simultaneously transferred to the athlete’s e-file. The same monitoring test was repeated at the end of the racing period. In case of a technical problem, such as the inability to communicate between the sensors and the mobile phone, the measurement was repeated the next day, as happened in one case. 

A primary target of the study was to assess the data quality issues on our mHealth monitoring. Thus, the data collection rate, performance of body sensors, quantity of data to be pre-processed and transferred (i.e., respecting data quote), as well as the quality of communication were evaluated. During the field effort, the participants were asked to report any of the experienced exercise-related symptoms, such as fatigue, dyspnea, inappropriate tachycardia, and dizziness. The reported symptoms were also automatically noted in the electronic file of each athlete. For the accurate evaluation of the m-health system measurements’ quality, the results of the two field monitoring tests and the athletes’ symptoms were correlated with the data from the pre-participation screening and the corresponding spiroergometric estimations (VO_2_max) in the laboratory. 

Descriptive statistics were used to describe categorical variables. Continuous variables were expressed as mean ± SD. The Shapiro–Wilk test was used for testing the normality of all data. The differences between values registered during the two testing periods were evaluated using the paired sample *t*-test. Relationships between categorical variables were tested using the Chi- Square statistic. For statistical analysis, the SPSS statistical program (Social Package for Social Sciences, Chicago, IL, USA, version 20.0) was used. A *p* < 0.05 was accepted as statistically significant.

## 3. Results

All 16 cyclists participated in all phases of the study. However, six athletes in each field training test interrupted their effort, due to the appearance of abnormal findings in mHealth monitoring. These were added in the results. All the cyclists had at least 5 years of racing experience, and they were trained more than five times per week. The demographic characteristics and the training habits of the participants are listed in Table 1. The data from the two maximal cardiopulmonary exercise tests, as well as from the two field monitoring tests, are presented in Table 2 and Table 3.

**Figure 2:**
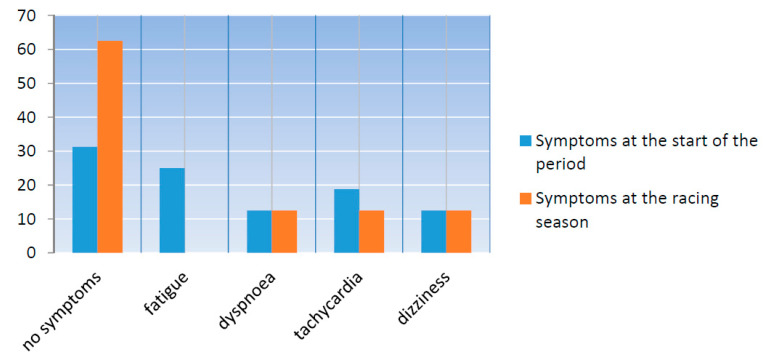
Percentage of athletes that reported health related symptoms during the two monitoring field tests.

From the first continuous field monitoring of cyclists’ physiological parameters, three cases of athletes’ peripheral capillary oxygen saturation of 88.5% were detected who also reported dyspnea, two cases of hypotension were detected who reported dizziness, and one case of paroxysmal supraventricular tachycardia was detected who reported palpitations at the beginning of the training period. Moreover, in the training period, field monitoring detected two athletes with hypotension appearing dizzy, two athletes with SpO_2_ 90% who reported dyspnea, and two athletes with extrasystoles in ECG describing palpitations (Figure 2).

One cyclist who developed hypotension was the same with a similar finding on the first measurement, an athlete who had a feeling of pallor had shown it during the initial evaluation, and an athlete with dyspnea had a decrease in O_2_ saturation during the first procedure. There were no correlations between the above findings and the medical history, clinical screening, and laboratory evaluation of the athletes. Specifically, the athletes with the abnormal symptoms from e-health monitoring do not have different values in VO2 max in comparison with the rest. All the athletes reported positive feedback for applying the e-health monitoring system, which gave them a feeling of security. All the athletes reported that the system was easy to use, and did not cause them problems in their movement. All the vital signs recorded via the monitoring system were legible, without noises. 

## 4. Discussion

The present study results show that the mHealth monitoring system could effectively and quickly detect health disorders during strenuous cycling training. This leads to a timely cessation of the athlete’s effort to prevent possible dangerous incidents. At the beginning of the racing season, training adaptations allowed the better management of the training load and less frequent health-related problems. Observed physiological changes were not significant, due to the elite level of the included cyclist and their long-term involvement in sport. 

e-Health monitoring, a modern approach in ambulatory health disorder detection and management of an athlete or a patient during exercise effort, has demonstrated the potential for considerable clinical utility in sports medicine [10]. Among other benefits, it provides ease of use; is relatively inexpensive; and is highly effective in supporting the diagnosis of various cardiac, metabolic, musculoskeletal, and other complications, especially those appearing during strenuous exercise [11]. A monitoring system based on an e-Health sensor board has been implemented before; it provides follow-up on athletes and measures vital physiological parameters [10,11,12]. In recent decades, many e-Health systems and applications have been developed in sports [10,11,12,16,17]. The devices in this class of monitoring can be divided into three categories: wearable-based, ambient-based (i.e., sensor-based approach), and camera-based (i.e., vision-based approach) [18]. Wearable detection approaches use sensors, such as accelerometers and gyroscopes, to detect and measure motion, location, and posture by measuring acceleration and orientation [19]. Ambient detection approaches use devices, such as pressure sensors, for movement detection [20]. They also rely on audio and vibration data analysis. The camera and vision detection approach, implemented in video tracking systems, relies on video data processing, such as activities in extreme conditions [21]. Wearable sensors have been embedded into watches, shirts, belts, etc. Such sensors provide real-time physiological information related to the health condition of the monitored subject. Various biosensors are used, such as electrocardiography sensors (ECG) used to monitor cardiac activity, electroencephalography sensors (EEG) used to monitor brain activity, electromyography sensors (EMG) used to monitor muscle activity, and electrooculography sensors (EOG) used to monitor eye movements [11,12]. Pulse oximeters are used to measure the oxygen level of the blood (i.e., oxygen saturation), while plethysmography sensors (EPG) are used to monitor the rate of blood flow [22]. Other biomedical parameters can also be evaluated using CO_2_ gas sensors to evaluate gaseous carbon dioxide levels to monitor respiration. Bluetooth communications were integrated to link the system to commercial medical devices for measuring blood pressure, blood glucose levels, etc. [11]. Data can be generated based on three event types: constant, interval, or instant [23]. Constant events ensure that data are continuously transmitted. The wearable sensors and mobile devices gathered biological signals and were connected to a personal digital assistant. The assistant held and processed biological signals and communicated with a computer server for additional processing, such as database services. 

A number of smart e-platforms have been proposed to monitor a lot of events in cycling [24,25,26]. The feedback provided by these platforms is however not sufficient in real-time efforts. Recently, the CONAMO project (CONtinuous Athlete MOnitoring), which combined the Wireless Personal-Area Network (WPAN) technology, widely used in cycling and the 6TiSCH network, has provided a dynamic, real-time, and reliable novel sensing system that can be used for covering cycling events, in amateur or professional cycling [25]. Gaidos and dos Sandos [26] designed and developed a system of mobile-Health monitoring and training for cyclists. The hardware application contained sensors for heart rate, oxygen percentage, speed, distance, time, and room temperature, during and after the training.

Our proposed mHealth monitoring system is very similar to many modern applications existing in smartphones. Using various sensors allowed exercising users to perform personal health checks based on their vital signs sent from sensors to the assistant. It can measure four parameters via biosensors (one lead ECG, heart rate, temperature, and respiratory rate) and the other four parameters (SPO_2_, blood pressure, glucose, and hemoglobin levels) by e-health application devices. Besides, it uses security to send the information and it could be implemented in teams of cyclists due to its easy scalability and cost-effectiveness. For the sports industry, wearable technology will always have extensive research in the microcontroller, power management of integrated circuits, and how sensor signal conditioning occurs. When these three are defined, the compatibility of sensory elements can be known for a required application, such as our mHealth system. Compared with other mobile health systems, our system has some advantages; it has a user-friendly operation process and lightweight on-body monitoring sensors. The physiologic parameters are measured by a wearable belt-like sensor and recorded by an Android smartphone via a specific software program, offering a real-time response for the abnormal situation. Such approaches provide several quality criteria to qualify data, such as accuracy, completeness, timeliness, relevance, legibility, accessibility, and usefulness [27]. In the previous studies [24,25] the sensors provided real-time feedback about the cyclists’ heart rate and heart rate training zones only, helping to coach and improving cycling experience and performance. The strength of our study was to investigate a wearable mHealth system to monitor a lot of physiological parameters of cyclists during intensive and strenuous training, in order to detect any health disorders early that may appear or progress during a race competition which demands an extreme body and mind effort. In comparison to a similar e-monitoring system for cyclists of Gaidos and Santos [26], there are some differences regarding the architecture of the mobile system as well as the ability to measure certain parameters. They monitored the saturation of oxygen; ambient temperature; and speed, distance, and altitude of the athlete. In addition, using the Karvonen formula, the maximum and minimum heart rate training range was calculated. Other researchers used a similar smart phone-based sensor interface technology to monitor some biological signals, specifically heart rate and body temperature during cycling, as well as riding and geographical information [28]. One of the results of the mHealth system developed in this project is the additional specialized examination of athletes that can lead to deeper insights, which are currently analyzed manually with wide margins of error. The results of biomedical information led to the conclusion that the developed system can be used to significantly increase speed and improve the quality and accuracy of monitoring during exercise. Finally, its management through the cloud facilitates its integration across platforms. Regarding data collection, the frequency of receiving data plays an essential role in overall system performance. 

Continuous field monitoring during the two field tests revealed arrhythmias, hypotension, and hypoxemia, which were confirmed by the athletes’ reported symptoms. Vigorous physical activity in susceptible individuals activates specific mechanisms that can lead to serious cardiac events [29,30] with cycling being the sporting activity with the highest number of adverse cardiac events according to a study by Vicent et al. [31]. Additionally, demanding endurance events followed by electrolyte imbalance and increased sympathetic activation in some cases can lead to transit myocardial ischemia and repolarization changes which are the ground base of severe arrhythmias [29]. Recent research by Breedt [1] and Killops et al. [4] concluded that medical complaints in cycling predominantly refer to sustained injuries which are followed by cardiovascular symptoms with the incidence of life-threatening medical encounters of 0.5 per 1000 athletes. Our study data showed that symptoms of increased fatigue and tachycardia appeared more commonly at the beginning of the training season indicating that the telemetric monitoring of elite cyclists should be considered from the start of the macrocycle. These performance-related and detraining-induced self-reported symptoms of fatigue and palpitations can be explained with literature data on injury and illnesses in armature cyclists [32,33]. Namely, these two studies showed that medical covering of recreational cycling events should consider heat, fatigue, and abdominal-related non-traumatic injuries. Dyspnea registered in our cyclists also at the beginning of the training season correlates with data from the study by McGrath et al., who found asthma and other respiratory-related symptoms to form 25% of all reported illnesses during mountain cycling events [34]. The results of the two CPETs also confirmed the high performance of our study’s participants, since the accomplished VO_2_max was above 55.4 mL/kg/min during the first assessment and 63.4 mL/kg/min during the second. These values are in agreement with the study by Buck and McNaughton [35], who found an average VO_2_max of 57.5 mL/kg/min in high-level cycling athletes. Notably, the above abnormal findings during mHealth monitoring at the fields were not expected, since the pre-participation screening of the cyclists at baseline did not reveal any pathological condition. Most of the athletes that had symptoms in their first attempt on the field had reappearing symptoms during their second effort. The above highlights the system’s usefulness in the early detection of disorders during the intense exercise of “healthy” athletes. However, we should emphasize that we were strict in our decision to interrupt the effort in some athletes with mild symptoms, because our primary concern in this experimental study was to assess the sensitivity of the mHealth system. Furthermore, our study data confirm the conclusion of a study on the medical coverage of cycling events that emphasizes the importance of communication systems between individual medical staff, race officials, and local emergency medical services [36].

The results of our study should be interpreted in light of some limitations. The main limitation is that only males and high-level cyclists participated in the study. Moreover, continuous monitoring was applied during an experimental strenuous cycling effort and not during a competitive cycling race, considering that the race-related stress may pose an additional health risk. 

## 5. Conclusions

In conclusion, the use of the novel described mHealth monitoring system in sports such as cycling was a feasible and usable method. Such monitoring during competitive cycling is an advantageous method for the early recording of cardiac and other health disorders of athletes, whose untimely evaluation could lead to unforeseen events. 

## Figures and Tables

**Table 1:** Cyclists’ demographic and training history data.

Age (years)	24.1 ± 4.5
Weight (Kg)	76.0 ± 6.9
Height (cm)	179.3 ± 5.2
ΒΜΙ	23.3 ± 1.5
Years of training (years)	5.6 ± 1.9
Frequency of training (times/week)	5.3 ± 0.9
Heart rate at rest (b/min)	56.2 ± 5.5
Systolic blood pressure at rest (mm/Hg)	125.8 ± 8.4
Diastolic blood pressure at rest (mm/Hg)	73.8 ± 10.0

**Table 2:** Results of the two maximal cardiopulmonary exercise tests.

Physiological Parameters	Beginning of Training Period	Racing Season
Heart Rate at rest (b/min)	71.4 ± 6.5	68.6 ± 8.7
Systolic Blood Pressure at rest (mm/Hg)	126.8 ± 7.1	125.8 ± 6.5
Diastolic Blood Pressure at rest (mm/Hg)	76.6 ± 9.4	75.7 ± 7.8
Heart Rate max (b/min)	195.7 ± 9.6	195.4 ± 10.0
Systolic Blood Pressure max (mm/Hg)	195.4 ± 6.4	194 ± 3.9
Diastolic Blood Pressure max (mm/Hg)	72.6 ± 2.9	71.8 ± 2.3
Time to fatigue (min)	11.69 ± 1.8	13.72 ± 1.3 *
Max Power (Watts)	354.7 ± 46.7	403.1 ± 30.1 *
HR_VT_ (b/min)	176.6 ± 8.7	175.6 ± 9.6
VO_2_max (mL/kg/min)	55.4 ± 5.3	63.4 ± 6.9 *

HR_VT_: Heart rate at the ventilatory threshold; VO_2_max: maximal oxygen consumption. * *p* < 0.05.

**Table 3:** Results of the two field monitoring tests.

Physiological Parameters	Start of the Training Period		Racing Season	
	Rest	Μax	Rest	Μax
Systolic Blood Pressure (mm/Hg)	131.2 ± 8.8	148.3 ± 13.6	130.8 ± 7.5	147.5 ± 8.3
Diastolic Blood Pressure (mm/Hg)	77.8 ± 9.4	76.3 ± 6.3	78 ± 8.5	77.8 ± 8.7
Heart Rate (b/min)	68.4 ± 6.4	165.6 ± 6.6	67.8 ± 6.2	163.6 ± 8.4
Hemoglobin (g/dL)	15.4 ± 1.5	14.5 ± 1.4 ^a^	15.2 ± 1.3	14.7 ± 1.2 ^b^
Hematocrit (%)	46.2 ± 4.5	43.4 ± 4.1 ^a^	45.5 ± 3.8	44.2 ± 3.8 ^b^
Glucose (mg/dL)	105.3 ± 8.4	100.7 ± 8.6 ^a^	99.8 ± 6.4 ^c^	94.8 ± 5.7 ^b^
Oxygen saturation	99.1 ± 0.7	96.4 ± 0.6 ^a^	98.7 ± 0.7 ^c^	96.9 ± 0.8 ^b^

^a^ *p* < 0.05 between rest and max in the preparation period; ^b^ *p* < 0.05 between rest and max in the racing season; ^c^ *p* < 0.05 between rest in the preparation period and in the racing season.

## Data Availability

The data presented in this study are available on request from the corresponding author. The data are not publicly available due to privacy of included athletes.
